# Clonal Spread of 16S rRNA Methyltransferase-Producing *Klebsiella pneumoniae* ST37 with High Prevalence of ESBLs from Companion Animals in China

**DOI:** 10.3389/fmicb.2017.00529

**Published:** 2017-03-29

**Authors:** Jing Xia, Liang-Xing Fang, Ke Cheng, Guo-Hao Xu, Xi-Ran Wang, Xiao-Ping Liao, Ya-Hong Liu, Jian Sun

**Affiliations:** ^1^National Risk Assessment Laboratory for Antimicrobial Resistance of Animal Original Bacteria, South China Agricultural UniversityGuangzhou, China; ^2^Guangdong Provincial Key Laboratory of Veterinary Pharmaceutics Development and Safety Evaluation, South China Agricultural UniversityGuangzhou, China; ^3^Jiangsu Co-Innovation Centre for Prevention and Control of Important Animal Infectious Diseases and ZoonosesYangzhou, China

**Keywords:** *Klebsiella pneumoniae*, 16S rRNA methyltransferases, companion animals, MLST

## Abstract

We screened 30 *Klebsiella pneumoniae* isolates from dogs and cats at a single animal hospital in Guangdong Province, China. Among them, 12 *K. pneumoniae* strains possessed high-level resistance to amikacin and gentamicin and these were screened for 16S rRNA methyltransferase (16S-RMTase) genes. And then the genes positive isolates were detected for ESBLs (extended spectrum β-lactamases) and analyzed by pulsed-field gel electrophoresis, multilocus sequence typing, PCR-based replicon typing and plasmid analysis. The genetic profiles of *rmtB* were also determined by PCR mapping. The twelve 16S-RMTase gene-positive isolates were *rmtB* (11/30) and *armA* (2/30) with one isolate carrying both genes. Extended spectrum β-lactamases genes were represented by *bla*_CTX-M-55_ (9/12), *bla*_CTX-M-27_ (2/12) and *bla*_CTX-M-14_ (1/12). The twelve 16S-RMTase containing strains were grouped into five clonal patterns and ST37 was the most prevalent sequence type. Ten *rmtB*-bearing plasmids conjugated successfully and all belonged to IncN and IncF (F33:A-:B-) incompatibility groups. Nine of the transconjugants carried a 97 kb plasmid and the other harbored both ∼60 and ∼200 kb plasmids. *rmtB* and *bla*_CTX-M-55_ were present on the same plasmid and indicated the co-transfer of these two genes, with the *rmtB* gene showing highly relevant relationships with IS*26* and Tn*3*. Our findings suggested a high prevalence of 16S-RMTase genes in *K. pneumonia* ST37 from dogs and cats. Additional studies are needed to trace the evolutionary path of this type of resistance among the *K. pneumonia* isolates, and to determine whether they have been transferred to humans.

## Introduction

*Klebsiella pneumoniae* is an opportunistic pathogen associated with a wide spectrum of community- and hospital-acquired infections (Lee et al., 2011). Increasing resistance to multiple antimicrobial agents has compromised the effectiveness of *K. pneumoniae* treatment options (Lee et al., 2011). Coexistence of resistance genes on the same plasmid appears to be the primary cause of the spread of resistance determinants (Luo et al., 2011). This has resulted in the appearance of multidrug resistant (MDR) or even pan drug-resistant (PDR) strains (Luo et al., 2011). Acquisition of MDR by Enterobacteriaceae members has become a global concern especially for *K. pneumonia* PDR strains (Zacharczuk et al., 2011a; Wasyl et al., 2015; Grevskott et al., 2017).

Aminoglycoside resistance is due primarily to aminoglycoside-modifying enzymes. Acetyltransferases, nucleotidyltransferases, and phosphotransferases inactivate commonly used aminoglycosides such as gentamicin (GEN) and tobramycin (Yang et al., 2011). Recently, plasmid-encoded 16S rRNA methyltransferases (16S-RMTase) have emerged in the Enterobacteriaceae family and in a group of glucose-non-fermentative microbes (Zacharczuk et al., 2011b). This is a new resistance mechanism to 4,6-disubstituted 2-deoxystreptamines and 4,5-disubstituted 2-deoxystreptamines. These structures encompass the majority of clinically important aminoglycosides (Zacharczuk et al., 2011b). Since the first report in 2003, ten 16S-RMTase-encoding genes, *rmtA*, *rmtB*, *rmtC*, *rmtD*, *rmtE*, *rmtF*, *rmtG*, *rmtH*, *armA*, and *npmA*, have been identified (O’Hara et al., 2013).

Reports on the prevalence of the 16S-RMTases have increased in the past years with the majority focused on the human clinical isolates including *K. pneumoniae* (Mezzatesta et al., 2013; Belbel et al., 2014; Bartoloni et al., 2016). Moreover, the majority of reports on dissemination of *armA* and *rmtB* to various Enterobacteriaceae species were focused on *E. coli* isolated from chickens and pigs (Yao et al., 2011; Yang Y. et al., 2015). Previous research has reported the occurrence of the ArmA methyltransferase in an ST11 clone of *K. pneumoniae* isolated from cats and dogs in Spain (Hidalgo et al., 2013a). Another research focused on the dissemination of *rmtB*-*bla*_CTX-M-9_ group genes and *rmtB*-*qepA* in Enterobacteriaceae isolates of dogs and cats in China mainly related to *E. coli* (Deng et al., 2011a). However, researches on prevalence and mechanisms of 16S-RMTase spread in pets via *K. pneumonia* strains in China are lacking.

Because of the common use of antimicrobial agents and the close contact with humans, companion animals may become potential sources for dissemination of antimicrobial resistance (Lloyd, 2007). Thus, we decided to identify the prevalence of 16S-RMTase genes among *K. pneumonia* isolates from dogs and cats in a veterinary hospital in Guangdong Province, China.

## Materials and Methods

### Bacterial Isolates

Thirty clinical *K. pneumoniae* strains were recovered from diseased dogs and cats during July, August, and September in 2010 and from July to October in 2012 at one animal hospital in Guangdong Province, China. Further information about the antimicrobial treatments of these pets were unfortunately not available. Samples were collected from the exudates of the infection areas (urinary tract infections, skin infections, or intra-abdominal infections) or feces, then seeded on MacConkey agar at 37°C. Each isolate was from a single animal. All bacterial species were identified with classical biochemical methods and confirmed using a matrix-assisted laser desorption and ionization time-of-flight mass spectrometry (MALDI-TOF MS) method (Shimadzu, Japan). As 16S-RMTase can confer high-level resistance to both GEN and amikacin (AMK), all isolates were subcultured in MacConkey agar containing 64 mg/L GEN and 64 mg/L AMK to screen for GEN/AMK-resistant isolates.

### Detection of Resistance Genes and Antimicrobial Susceptibility Testing

All the strains exhibiting aminoglycoside resistance were screened for 16S-RMTase genes including *rmtA*, *rmtB*, *rmtC*, *rmtD*, *rmtE*, *rmt*F, *rmtG*, *rmtH*, *armA*, and *npmA* (Hidalgo et al., 2013b; Xia et al., 2016). Aminoglycoside-resistant isolates were further analyzed for the presence of extended spectrum β-lactamase (ESBL) genes (*bla*_TEM_, *bla*_CTX-M_, and *bla*_SHV_) using previously described primers (Liao et al., 2015).

The minimal inhibitory concentration (MIC) of ampicillin (AMP), cefotaxime (CTX), meropenem, ciprofloxacin (CIP), norfloxacin (NOR), GEN, kanamycin (KAN), AMK, streptomycin (STR), neomycin (NEO), apramycin (APR), chloramphenicol (CHL), florfenicol (FFC), tetracycline (TET), sulfamethoxazole/trimethoprim (SXT) were determined by the agar dilution method according to Clinical and Laboratory Standards Institute guidelines (Clinical and Laboratory Standards Institute, 2015). *E. coli* ATCC 25922 was used as a quality control strain.

### Clonal Relatedness

Chromosomal DNA digested with *Xba*I restriction enzyme was used for pulsed-field gel electrophoresis (PFGE) to analyze the genetic relatedness of all isolates containing 16S-RMTase genes (CHEF Mapper1, Bio-Rad Laboratories, Hercules, CA, USA) as previously described (Fang et al., 2015). PFGE patterns were analyzed with the Dice coefficient and the unweighted pair group method with average linkages (UPGMA) clustering method using BioNumerics software (Applied Maths, Ghent, Belgium). PFGE types were defined with >90% similarity between clusters. Multilocus sequence typing (MLST) of 16S-RMTase genes containing *K. pneumoniae* was performed according to the protocols described on the *K. pneumoniae* database website^1^. Seven chromosomal genes were PCR amplified and sequenced. Then the sequences were compared with those reference sequences, submitted to the MLST database to determine allele numbers and STs (Belbel et al., 2014).

### Transfer of the 16S-RMTase Genes and Plasmids Replicon Typing

Isolates positive for 16S-RMTase genes were selected for conjugation experiments by the broth-mating method using STR-resistant *E. coli* C600 as the recipient to determine the transferability of 16S-RMTase genes. Transconjugants were selected on MacConkey agar plates supplemented with STR (1000 μg/mL) and AMK (64 μg/mL). Transconjugants harboring 16S-RMTase genes were confirmed by PCR and antimicrobial susceptibility testing as described above (Fang et al., 2015). Antimicrobial susceptibility testing of the transconjugants and co-transfer of other resistance genes as mentioned above were also determined.

Plasmids were preliminarily classified according to their incompatibility group by using the PCR-based replicon typing (PBRT) scheme described previously (Yao et al., 2011). To better characterize IncF plasmids, replicon sequence typing of IncF plasmids was performed according to the protocol previously described (Yang Q.E. et al., 2015). The A- and B- symbols indicate the absence of the FIA and FIB replicons, respectively.

### Plasmid Analysis

PFGE with S1 nuclease (TakaRa Biotechnology, Dalian, China) digestion of genomic DNA was performed for all the transconjugants as described previously (Xia et al., 2015). After Southern blotting, fix the DNA to a Hybond-N^+^ membrane (GE Healthcare, Little Chalfont, UK), the plasmids were probed with the 16S-RMTase genes and *bla*_CTX-M-1G/9G_ gene (DIG High Prime DNA Labeling and Detection Starter Kit I, Roche Applied Science, Mannheim, Germany). All the transconjugants were subjected to restriction enzyme digestion (*Eco*RI) analysis to clarify whether a specific plasmid had been disseminated among the isolates (Deng et al., 2011b). *Eco*RI-RFLP types were defined with >80% similarity between clusters using the method described as PFGE analysis.

### Genetic Profiles of 16S-RMTase Genes

Reports on the genetic contexts of the 16S-RMTase genes indicated that IS*26*, Tn*3*, IS*CR1*, and IS*CR3* played an important role in the dissemination of these genes. These transfer events enabled horizontal spread among different bacterial lineages (Deng et al., 2011b). Here we focused on the link between 16S-RMTase genes and mobile elements by PCR mapping. The primers using in PCR mapping were listed in Supplementary Table S1.

### Ethics Statement

In this study, the owners of the companion animals from which fecal swabs and the exudates of the infection areas were taken gave permission for their animals to be used in this study. Written informed consent was obtained, and the study protocol in our research was approved by the South China Agriculture University Animal ethics committee and carried out in accordance with relevant guidelines.

## Results

### Prevalence of 16S-RMTase Genes and Antimicrobial Susceptibility Testing

Among 30 *K. pneumoniae* isolates from the hospital, 12 were resistant to AMK and GEN. However, all *K. pneumoniae* strains contained the 16S-RMTase genes *rmtB* (11/30) and/or *armA* (2/30) and a single isolate carried both genes (1/30). The AMK/GEN-resistant *K. pneumoniae* isolates showed an MDR phenotype (resistant to three or more classes of antimicrobials). Apart from aminoglycoside antibiotics, there was a very high frequency (>90.0%) of resistance to AMP, CTX, SXT, TET, CIP, NOR, CHL, and FFC. The most frequently observed pattern of MDR was GEN-CTX-SXT-AMP-CIP-TET-STR-KAN-AMK-CHL-NEO-NOR (**Table 1**).

**Table 1:** 16S rRNA methyltransferases gene-positive *K. pneumoniae* isolates from this study.

Strains^a^	Origin	Year	Resistance profiles^b^	Resistant genes^c^	MLST	Replicon types^d^	Plasmids size (kb)
KP01	Dog	2010	GEN, CTX, SXT, AMP, CIP, TET, STR, KAN, AMK, CHL, NEO, NOR	*rmtB, armA*, *bla*_TEM-1,_ *bla*_SHV -1_	ST2018	NT	60 (*rmtB*), 200 (*armA*)
KP04	Cat	2010	GEN, SXT, AMP, FFC, CIP, TET, KAN, AMK, CHL, NEO, APR, NOR	*armA*	ST395	ND	ND
KP07	Dog	2012	GEN, CTX, SXT, AMP, FFC, CIP, TET, STR, KAN, AMK, CHL, NEO, NOR	*rmtB*, *bla*_TEM-1_, *bla*_CTX-M-55_, *bla*_SHV -1_	ST37	F33:A-:B-, N	97
KP16	Cat	2012	GEN, CTX, SXT, AMP, FFC, CIP, TET, STR, KAN, AMK, CHL, NEO, APR, NOR	*rmtB*, *bla*_SHV -1_	ST147	ND	ND
KP21	Dog	2012	GEN, CTX, SXT, AMP, FFC, CIP, TET, STR, KAN, AMK, CHL, NEO, NOR	*rmtB*, *bla*_TEM-1_, *bla*_CTX-M-55,_ *bla*_SHV -1_	ST37	F33:A-:B-, N	97
KP22	Cat	2012	GEN, CTX, SXT, AMP, FFC, CIP, TET, STR, KAN, AMK, CHL, NEO, NOR	*rmtB*, *bla*_TEM-1_, *bla*_CTX-M-55,_ *bla*_SHV -1_	ST37	F33:A-:B-, N	97
KP23	Dog	2012	GEN, CTX, SXT, AMP, FFC, CIP, TET, STR, KAN, AMK, CHL, NEO, NOR	*rmtB*, *bla*_TEM-1_, *bla*_CTX-M-55,_ *bla*_CTX-M-27,_ *bla*_SHV -1_	ST37	F33:A-:B-, N	97
KP24	Dog	2012	GEN, CTX, SXT, AMP, FFC, CIP, TET, STR, KAN, AMK, CHL, NEO, NOR	*rmtB*, *bla*_TEM-1_, *bla*_CTX-M-55,_ *bla*_CTX-M-27_, *bla*_SHV -1_	ST37	F33:A-:B-, N	97
KP25	Dog	2012	GEN, CTX, SXT, AMP, FFC, CIP, TET, STR, KAN, AMK, CHL, NEO, NOR	*rmtB*, *bla*_TEM-1_, *bla*_CTX-M-55,_ *bla*_SHV -1_	ST37	F33:A-:B-, N	97
KP26	Dog	2012	GEN, CTX, SXT, AMP, FFC, CIP, TET, STR, KAN, AMK, CHL, NEO, NOR	*rmtB*, *bla*_TEM-1_, *bla*_CTX-M-55,_ *bla*_CTX-M-14_, *bla*_SHV -1_	ST37	F33:A-:B-, N	97
KP29	Dog	2012	GEN, CTX, SXT, AMP, FFC, CIP, TET, STR, KAN, AMK, CHL, NEO, NOR	*rmtB*, *bla*_TEM-1_, *bla*_CTX-M-55_	ST37	F33:A-:B-, N	97
KP30	Dog	2012	GEN, CTX, SXT, AMP, FFC, CIP, TET, STR, KAN, AMK, CHL, NEO, NOR	*rmtB*, *bla*_TEM-1_, *bla*_CTX-M-55,_ *bla*_SHV -1_	ST37	F33:A-:B-, N	97

^a^*Isolates with transconjugants are underlined.*

^b^*Resistance phenotypes transferred to the recipient by conjugation are underlined. GEN, gentamicin; CTX, cefotaxime; SXT, sulfamethoxazole/trimethoprim; AMP, ampicillin; FFC, florfenicol; AMK, amikacin; CIP, ciprofloxacin; TET, tetracycline; STR, streptomycin; KAN, kanamycin; CHL, chloramphenicol; NEO, neomycin; APR, apramycin; NOR, norfloxacin.*

^c^*Genes that were co-transferred by conjugation are underlined.*

^d^*ND, not determined; NT, non-typeable*.

### Characterization of Resistance Genes

We examined the prevalence of ESBL and AME genes in the 16S-RMTase gene-positive isolates. Among these, nine isolates contained *bla*_CTX-M_ that were distributed as *bla*_CTX-M-55_ (6), *bla*_CTX-M-55_ and *bla*_CTX-M-27_ (2), and *bla*_CTX-M-55_ and *bla*_CTX-M-14_ (1). Two other ESBLs were also present in these isolates as *bla*_TEM-1_ (10/12) and *bla*_SHV -1_ (10/12) (**Table 1**).

### PFGE Typing and MLST

**FIGURE 1:**
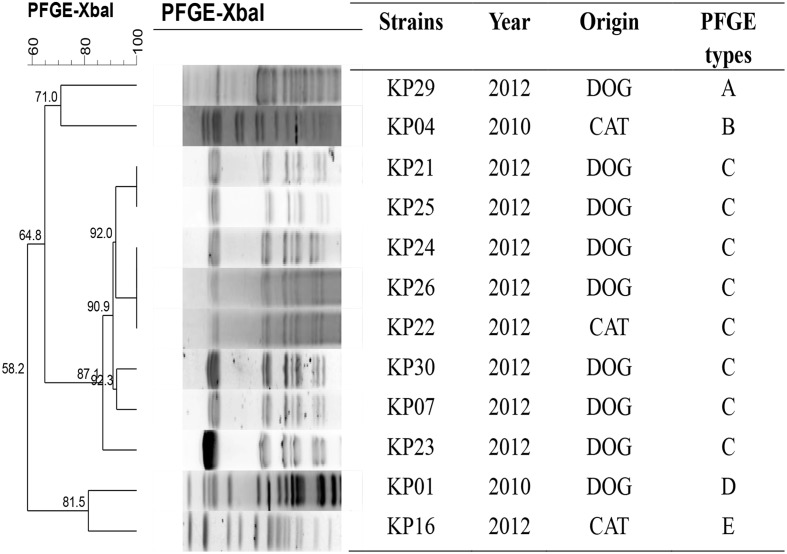
**PFGE analysis of the twelve 16S-RMTase-positive *K. pneumoniae* isolates**.

PFGE was successfully performed in all twelve 16S-RMTase genes positive *K. pneumoniae* isolates and were grouped into five clonal patterns designated PFGE types A to E (**Figure 1**). The predominant PFGE type was type C that accounted for 67% (8/12) of the isolates, suggesting that one small clonal outbreak had occurred. The MLST results revealed that one ST was the most prevalent in this collection: ST37 (9/12). ST2018, ST395, and ST147 each had one isolate (**Table 1**). The results obtained by MLST were consistent with those obtained by PFGE; eight *rmtB*-positive *K. pneumoniae* strains showing similar PFGE patterns were belonged to ST37.

### Transfer of the 16S-RMTase Genes and Plasmid Analysis

Plasmids carrying *rmtB* from 10 isolates were successfully transferred to recipients by conjugation. All the transconjugants were highly resistant to AMK, GEN, and KAN (MICs ≥ 512 μg/mL). PBRT of the plasmid incompatibility groups showed that nine *rmtB*-bearing plasmids from *K. pneumoniae* isolates carried two replicons. The two replicons were confirmed to be IncFII in combination with IncN, and the other *rmtB*-bearing plasmid was non-typeable. All the IncFII plasmids were further determined to be F33:A-:B-.

**FIGURE 2:**
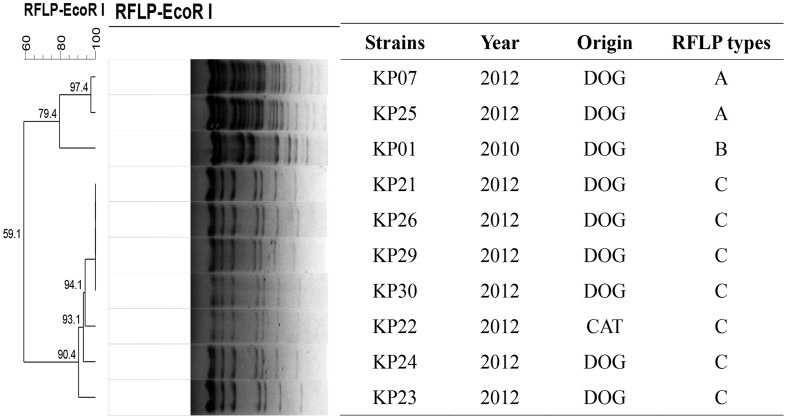
***Eco*RI-RFLP analysis of the 10 transconjugants in the 16S-RMTase-positive *K. pneumoniae* isolates**.

Nine of the 10 transconjugants carried one approximately 97 kb plasmid. The other one harbored both ∼60 kb (*rmtB* positive) and ∼200 kb (*armA* positive) plasmids at the same time. Moreover, *rmtB* and *bla*_CTX-M-55_ genes were located on the same 97 kb plasmid. The RFLP analysis indicated that 7 out of the 10 transconjugants had identical plasmid restriction patterns. The two remaining plasmids shared the same RFLP type (**Figure 2**).

### Genetic Profile Analysis

**FIGURE 3:**
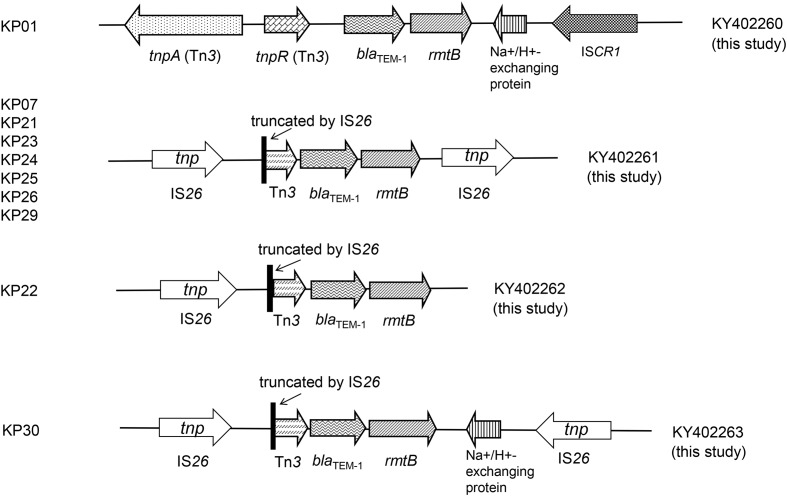
**Genetic profiles of the *rmtB* gene**.

The genetic profiles were successfully obtained in the 10 *rmtB*-positive transconjugants. We found four new genetic profiles in the 10 *rmtB*-positive transconjugants and all possessed Tn*3* and *bla*_TEM-1_ directly upstream of *rmtB*. Tn*3* was truncated by IS*26* in 9 of the 10 transconjugants, which were inserted at the same site (3573 of Tn*3*). The other isolate KP01 harbored the intact *tnpA*–*tnpR* genetic structure of Tn*3*, with a Na^+^/H^+^-exchanging protein gene and IS*CR1* downstream *rmtB*. IS*26* was also found directly downstream of *rmtB* in seven plasmids, which was located in the same orientation to the 5′ IS*26* elements. Moreover, the Na^+^/H^+^-exchanging protein gene located between *rmtB* and IS*26* (in the opposite orientation) occurred in strain KP30 plasmid (**Figure 3**). The four unique sequences were deposited in GenBank under accession numbers KY402260, KY402261, KY402262, and KY402263.

## Discussion

The 16S-RMTase genes confer broad and high-level resistance to most clinically available aminoglycosides (Habeeb et al., 2013). This has become a worldwide concern especially when these genes are combined with other classes of antimicrobial resistance determinants. This has led to the emergence of MDR strains (Habeeb et al., 2013).

In the present study, our results highlighted a high prevalence of 16S-RMTase genes in *K. pneumoniae* isolates from pets compared with other reports of human infections (Yan et al., 2004; Yu et al., 2009). Similarly, these 16S-RMTase-positive *K. pneumoniae* isolates were all MDR strains and mostly resistant to β-lactams, aminoglycosides, TETs, amphenicols, sulfonamides, and fluoroquinolones. Fortunately, the isolates we investigated in this study were susceptible to carbapenem antibiotics, which was different from other studies (Zacharczuk et al., 2011b; Li et al., 2012).

The twelve 16S-RMTase-positive *K. pneumoniae* isolates were successfully typed into five different groups by PFGE. Group C predominated and contained eight isolates indicating that the *rmtB*-positive isolates were spread by clonal dissemination. MLST analysis indicated four STs: ST37, ST2018, ST395, and ST147. The most prevalent ST in this collection was ST37 containing eight isolates belonging to the same PFGE type C group.

Previous reports had documented that ST11 was the most prevalent 16S-RMTase-positive *K. pneumoniae* in human isolates. ST101 was the second frequently reported ST type, which often co-harbored KPC-2 (*K. pneumoniae* carbapenemase) in 16S-RMTase-positive *K. pneumoniae* (Li et al., 2012; Mezzatesta et al., 2013; Hu et al., 2014; Seiffert et al., 2014). However, the sharing of ST37 between isolates in our study is important because KPC-producing *K. pneumoniae* ST37 of human origin has been detected only sporadically in China (Yang et al., 2013; Liu et al., 2015). This observation might provide further support in concerns about transmission of resistance between companion animals and humans.

ESBL genes, especially CTX-M genes, frequently coexist and co-transfer with 16S-RMTase genes in Enterobacteriaceae members including *K. pneumoniae* (Yan et al., 2004; Bogaerts et al., 2007; Zacharczuk et al., 2011a). In this study, PCR and sequence analyses revealed that *bla*_CTX-M-55_ and *bla*_TEM-1_ were co-transferred with *rmtB* in 9 and 10 transconjugants of *K. pneumoniae* strains, respectively. This confirmed an intimate association between ESBLs and 16S-RMTase genes (Deng et al., 2011a; Xia et al., 2016). In addition, PCR-based plasmid replicon typing indicated that the nine transconjugants were in the FII and N incompatibility groups. All IncFII plasmids belonged to the same F33:A-:B- subgroup. This finding is similar with reports of *rmtB*-positive *E. coli* from food animals, pets and humans in China (Yu et al., 2010; Deng et al., 2011a,b; Yao et al., 2011; Xia et al., 2016).

This data indicated the horizontal transmission of the prevailing plasmids from variety bacterial species between different hosts. Moreover, our plasmid digestion profiles and S1-PFGE analysis confirmed that the prevalence of the *rmtB*-positive *K. pneumoniae* isolates was due to the dissemination of a ∼97 kb plasmid containing *bla*_CTX-M-55_.

Previous studies indicated that Tn*3*, IS*26* and IS*CR3* were associated with the transmission of *rmtB* (Doi and Arakawa, 2007; Deng et al., 2011b). In this study, the *rmtB* gene showed highly relevant relationships with IS*26* and Tn*3*. The presence of a truncated Tn*3* inserted *via* IS*26* upstream of *rmtB*, and IS*26* inserting in the same direction located downstream of *rmtB*, was the most prevalent structure in seven transconjugants. Within this group, five plasmids of the corresponding transconjugants shared similar *Eco*RI-RFLP patterns (**Figures 2**, **3**). This indicated a plasmid with this particular genetic profile was the origin of *rmtB* in the *K. pneumoniae* isolates from this group of pets.

In conclusion, *rmtB* was the most prevalent 16S-RMTase gene of *K. pneumoniae* that originated in pets. The dissemination of *rmtB*-producing *K. pneumoniae* isolates in this study appeared to be clonal spread and horizontal transfer. ST37 *K. pneumoniae* and F33:A-:B- plasmids sized ∼97 kb with the same genetic profile mediated by IS*26* transmission both contributed to the dissemination of *rmtB*. In this study, we investigated only a single veterinary hospital. An organized surveillance effort is required to better understand the scope of this problem and identify control measures among hospitals.

## Author Contributions

Y-HL and X-PL designed and organized the study. JX and L-XF did the research. KC, G-HX, and X-RW did the assisted help. JS analyzed the data and wrote the paper.

## Conflict of Interest Statement

The authors declare that the research was conducted in the absence of any commercial or financial relationships that could be construed as a potential conflict of interest.

## Footnotes

1 http://bigsdb.pasteur.fr/klebsiella/
