# Supplementary material for: Clonal Spread of 16S rRNA Methyltransferase-Producing Klebsiella pneumoniae ST37 with High Prevalence of ESBLs from Companion Animals in China
Source: Front Microbiol. 2017 Mar 29;8:529. doi: 10.3389/fmicb.2017.00529 (PMC5389360; doi:10.3389/fmicb.2017.00529)
Supplement: Supplementary file 1 [file Table_1.DOCX]

**Table S1. Primers used for PCR mapping in this study**

| **Genes** | **Primers** |
| --- | --- |
| IS*CR1* -F | GTC AAT CGC CCA CTC AAA C |
| IS*CR1* -R | TCT TCG GCA TAG ACA CCA TC |
| IS*CR3* -F | GGT CGG CGG CTT CTC ACT G |
| IS*CR3* -R | GCA CGC AGG TTT GCA TTC G |
| Tn*3*-F | ATC AGT GCC AGT TTG TTC C |
| Tn*3*-R | ACC TGC GTC TTT GAG TGC |
| IS*26*-F | ATG AAC CCA TTC AAA GGC CG |
| IS*26*-R | TTA CAT TTC AAA AAC TCT GCT TAC C |
